# Exercise inhibits JNK pathway activation and lipotoxicity *via* macrophage migration inhibitory factor in nonalcoholic fatty liver disease

**DOI:** 10.3389/fendo.2022.961231

**Published:** 2022-09-06

**Authors:** Ni Cui, Hui Li, Yaoshan Dun, Jeffrey W. Ripley-Gonzalez, Baiyang You, Dezhao Li, Yuan Liu, Ling Qiu, Cui Li, Suixin Liu

**Affiliations:** ^1^Division of Cardiac Rehabilitation, Department of Physical Medicine and Rehabilitation, Xiangya Hospital of Central South University, Changsha, China; ^2^National Clinical Research Center for Geriatric Disorders, Xiangya Hospital of Central South University, Changsha, China; ^3^Division of Preventive Cardiology, Department of Cardiovascular Medicine, Mayo Clinic, Rochester, MN, United States

**Keywords:** exercise, NAFLD (non alcoholic fatty liver disease), MIF — macrophage migration inhibitory factor, lipotocixity, JNK

## Abstract

The macrophage migration inhibitory factor (MIF) expressed in hepatocytes can limit steatosis during obesity. Lipotoxicity in nonalcoholic fatty liver disease is mediated in part by the activation of the stress kinase JNK, but whether MIF modulates JNK in lipotoxicity is unknown. In this study, we investigated the role of MIF in regulating JNK activation and high-fat fostered liver lipotoxicity during simultaneous exercise treatment. Fifteen mice were equally divided into three groups: normal diet, high-fat diet, and high-fat and exercise groups. High-fat feeding for extended periods elicited evident hyperlipemia, liver steatosis, and cell apoptosis in mice, with inhibited MIF and activated downstream MAPK kinase 4 phosphorylation and JNK. These effects were then reversed following prescribed swimming exercise, indicating that the advent of exercise could prevent liver lipotoxicity induced by lipid overload and might correlate to the action of modulating MIF and its downstream JNK pathway. Similar detrimental effects of lipotoxicity were observed in *in vitro* HepG2 cells palmitic acid treatment. Suppressed JNK reduced the hepatocyte lipotoxicity by regulating the BCL family, and the excess JNK activation could also be attenuated through MIF supplementation or exacerbated by MIF siRNA administration. The results found suggest that exercise reduces lipotoxicity and inhibits JNK activation by modulating endogenous hepatic MIF in NAFLD. These findings have clinical implications for the prevention and intervention of patients with immoderate diet evoked NAFLD.

## Introduction

Nonalcoholic fatty liver disease (NAFLD) is an amalgamation of heterogenous liver diseases including liver pathologies such as steatosis, steatohepatitis, fibrosis, and cirrhosis. It is characterized by the aggregation of different lipid species and derivatives within hepatocytes. Within NAFLD pathophysiology, hepatocyte lipotoxicity is recognized as one of the key events. Lipotoxicity is defined as cellular dysfunction resulting from the excessive and ectopic accumulation of lipids in cells (1). The consequent hepatocellular injury or apoptosis is associated with toxic lipid accumulation as a result of the hyper-alimentation diets rich in lipids and carbohydrates. Lipotoxicity is thought to be a contributor to metabolic inflexibility with JNK as a major effector of this process (2). JNK is a dominant effector mitogen activated protein kinase (MAPK) that arises in response to physical and chemical stress, including nutrient alterations contributing to lipotoxicity (3), and is primarily mediated by its upstream enzyme, MAPK kinase 4 (MKK4) (4). It has been suggested that JNK aggravates liver steatosis by reducing free fatty acid (FFA) oxidation in liver cells (5), directly stimulates the hepatocyte apoptosis pathway, and acts on the Bcl2 family to trigger death (6).

The macrophage migration inhibitory factor (MIF) is an immunoregulatory mediator expressed in tissues and involved in the modulation of dysmetabolic effects including insulin resistance (7), glucose, and FFA metabolism (8). In the liver, MIF is primarily produced by hepatocytes and satellite cells (9). The action of MIF signals interacting with the HLA class II histocompatibility antigen (CD74) receptor mitigates the steatosis of liver cells (10). The MIF also reduces liver fibrosis during chronic liver injury in mice dependent on CD74 (11). Further research has provided evidence that MIF-based suppression of apoptosis is mediated through the modulation of MKK4 upstream kinases (MKKKs) (12) (13) and JNK activity (14) in cardiomyocytes and pulmonary endothelial cells; however, whether this is the case in liver cells remains without certainty.

Exercise exerts ameliorating effects on obesity and NAFLD. Exercise counteracts lipotoxicity by improving lipid turnover in skeletal muscle (1) and limiting lipid metabolic disorder in the prevention of myocardial lipotoxicity (15). The effect of exercise on NAFLD might be closely related to the MIF signal axis. Hyo et al. suggested that 4-week treadmill exercise significantly increased hepatic MIF expression in normal mice and protected against liver steatosis (16). In addition, exercise may inhibit JNK activation by regulating P53 and delaying the transformation of hepatocytes into carcinoma in obese mice (17). Our previous findings indicated that aerobic exercise enhanced lipid metabolism and reduced lipid droplet accumulation and liver steatosis in NAFLD mice (18). However, what is not yet known are the effects of exercise on lipotoxicity and, in particular, how this fits within the MIF signaling pathway in NAFLD.

Therefore, this study aims to investigate MIF as a potential therapeutic target for regulating lipotoxicity in NAFLD. We hypothesize that exercise can modulate endogenous hepatic MIF by inhibiting JNK activation after lipid accumulation and limiting cell apoptosis.

## Materials and methods

### Study approval

All experiments involving mice were conducted following the Guide for the Use of Laboratory Animals Animal Welfare Legislation of China, as well as the “Animal exercise studies in cardiovascular research: Current knowledge and optimal design—A position paper of the Committee on Cardiac Rehabilitation, Chinese Medical Doctors’ Association and the Animal Ethics Committee of Xiangya Medical School, Central South University” (approval ID: SYXK 2015-0017). Every effort was made to minimize any unnecessary discomfort to the animals.

### Animals and study design

Fifteen male C57BL mice (8 weeks of age, 18 ± 2 g) were used in this study (19); these were housed in individually ventilated cages at room temperature (22 ± 2°C), with free access to water and food and an artificial 12-h light/dark cycle. The mice were randomly divided into three equal-sized groups (*n* = 5): the normal diet feeding group (ND, 18% of total calories from fat), the high-fat feeding group (HFD) (HF, 45% of total calories from fat), and the high-fat diet + exercise group (EX, moderate-intensity swim training).

Mice undertaking exercise did so under an adaptive swimming protocol. During the first week, training duration was set as 10 min, gradually increasing by 10 min per day until a final level of 60 min per session was reached. After an adaptation period was completed, the exercise group was given a high-fat feeding diet and 60-min swim training in a pool (diameter, 120 cm; high, 60 cm; water depth, 30 cm; temperature, 30 ± 2°C). The activity area of each mouse during swimming exceeded 200 cm (2). Following each session, the mice were quickly pulled out of the water, wiped and dried with a towel, and then put back into the cage. Swimming sessions were conducted between 9 am and 2 pm each day and continued for 5 days/week for 16 consecutive weeks. The other two groups did not receive swimming training. After 16 weeks, the mice were sacrificed for liver tissue and serum collection following 12-h fasting.

### Immunohistochemistry

Liver tissues were removed, fixed in 4% paraformaldehyde, then deparaffinized and processed through a series of increasing concentrations of ethanol for dehydration. These samples were subsequently paraffin-embedded and sectioned. The sections were then stained with hematoxylin and eosin for general histological assessment or the immunohistochemical analysis of anti-MIF primary antibody (20). Slides were subjected to 20 min of autoclave heating (0.01 Mcitrate buffer, pH 6.0) for antigen retrieval. After blockage of the endogenous activity of peroxidase by incubation with 1% periodic acid for 10 min, sections were then incubated with anti-MIF antibody (ab187064, Abcam) overnight at 4°C, followed by 30 min of incubation with secondary antibodies. Slides were finally visualized by diaminobenzidine (DAB) Chromogen for 5 min. After hematoxylin staining of the nucleus, all pieces were sealed with neutral gum and evaluated using a microscope (BA410T, Motic). The positive criterion was MIF showing fine, granular dark-brown depositions. Lipid accumulation was quantified by the area of lipid droplets (LDs) in liver tissues *via* Oil Red O (Wellbio, Changsha, China) staining. The staining images were analyzed by using ImageJ software.

### TUNEL analysis

To detect cell apoptosis, terminal deoxynucleotidyl transferase-mediated 2′-deoxyuridine 5′-triphosphate nick-end labeling (TUNEL) assay staining was performed following manufacturers’ instructions (21). Embedded frozen liver tissue sections were prepared and stained with TUNEL reagents after sequential deparaffinization followed by an apoptosis *in situ* detection kit (Yeasen Biotechnology, Shanghai, China). 4′,6-diamidino-2-phenylindole (DAPI) staining was used to visualize the nuclei. TUNEL-positive cells labeled with fluorescein isothiocyanate were imaged *via* fluorescence microscopy (BA410T, Motic). The frequency of apoptotic cells in the liver section was semi-quantified by determining the percentage of TUNEL-positive cells in three microscopic fields per specimen.

### Biochemical analyses

The total cholesterol (TC, A111-2-1), triglycerides (TG, A110-1-1), nonesterified-free fatty acids (NEFA, A042-1-1 A042-2-1), MDA (A003-1-2), and CuZn-SOD activity (A001-2-2) levels were determined in tissues and hepatocytes by using commercial kits according to the manufacturer’s instructions (Jiancheng Bioengineering Institute, Nanjing, Jiangsu, China).

### RNA and protein analysis

The mRNA and protein analyses were performed in tissue samples and cultured cells. Transcript levels for the genes MIF, CD74, FAS, SREBP-1c, SCD1, ACOX1, and CD36 were measured by quantitative real-time reverse transcription PCR (qRT-PCR) phosphorylation. The total mRNA was isolated using an RNA extraction kit (Trizol, Thermo, Waltham, Massachusetts, USA) and synthesized cDNA using a HiFiScript cDNA Synthesis Kit (Cwbiotech, Beijing, China). SYBR Green (Cwbiotech, Beijing, China) was used to quantify the PCR amplification products. The total levels of MKK-4 and JNK and total MIF, BAD, BAX, and Bcl2 content were evaluated by Western blot as previously described (19). The primers and antibodies used are listed in the **Supplementary Table**. The values of genes were normalized to the actin levels.

#### Cell culture

Human hepatocellular carcinoma HepG2 cells were plated on black 96-well plates with clear bottoms at 1 × 10 (4) cells per well in Dulbecco Modified Eagle Medium (DMEM) containing 10% fetal bovine serum (FBS) and 1% antibiotics at 37°C, 5% CO_2_. After 24 h, 60–80% of the confluent cells were treated with palmitate acid (PA) (P5585-10G, Sigma-Aldrich, St. Louis, Missouri, USA) to build an NAFLD cell model. A total of 153.9 mg PA was dissolved in dimethylsulfoxide (1.5 ml) and prepared at concentrations of 0, 200, 400, and 800 μm, respectively, to test toxicity. Further experiments were performed to determine the decent concentration. We found that, with PA concentrations from 400 nm to 800 μm, the viability rates declined; therefore, the dosage of PA was set at 400 μm.

### RNA interference and MIF reagents

DMEM (95 μl) and small-interfering RNA targeting MIF (siMIF) or JNK (siJNK) (5 μl) were mixed. The cells were transfected with siRNA (si-MIF or si-JNK) for 24 h using Lipofectamine 2000 with serum-free media, then treated with 0.4 mM BSA, 0.4 mM palmitate, and 1 mM rapamycin in a serum-free medium for 24 h. rMIF (200 ng/ml) was then added for a further 24 h. MIF and JNK siRNAs were purchased from RiboBio (Guangzhou, China) and Lipofectamine 2000 from Invitrogen (Carlsbad, CA, USA). Recombinant mouse MIF (rMIF) was purchased from PeproTech (300-69-25). Further experiments were performed. rMTF (25 μg) was dissolved in phosphate buffer saline (PBS) and prepared to the original concentration of 200 μg/ml. Then, 2 μl of the original solution was added into 1,999 μl of complete medium balance to 200 ng/ml and diluted into concentrations of 25, 50, 100, and 200 ng/ml when in use. Doses of rMIF were chosen to refer to previous publications (10 11).

### Flow cytometry

HepG2 cells were collected by trypsin without ethylenediaminetetraacetic acid (EDTA) followed by washing in PBS. Cells (3.2 × 105) were obtained and suspended in 500 μl of 1× annexin V–binding buffer blended with annexin V–APC (5 μl) and propidium iodide (PI) (5 μl) (KeyGen Biotech, JiangSu, China) and then incubated in the absence of light for 10 min. The results were evaluated immediately through flow cytometry (BD FACSCanto, Franklin Lakes, New Jersey, USA) (22).

### Statistical analysis

One-way ANOVA with Student–Newman–Keuls tests was used to determine the differences between group mean values. *P* < 0.05 was considered statistically significant. Values are displayed as *M* ± SEM.

## Results

### Exercise improves lipid profiles and alleviates the lipid accumulation in mice fed with a high-fat diet

**Figure 1:**
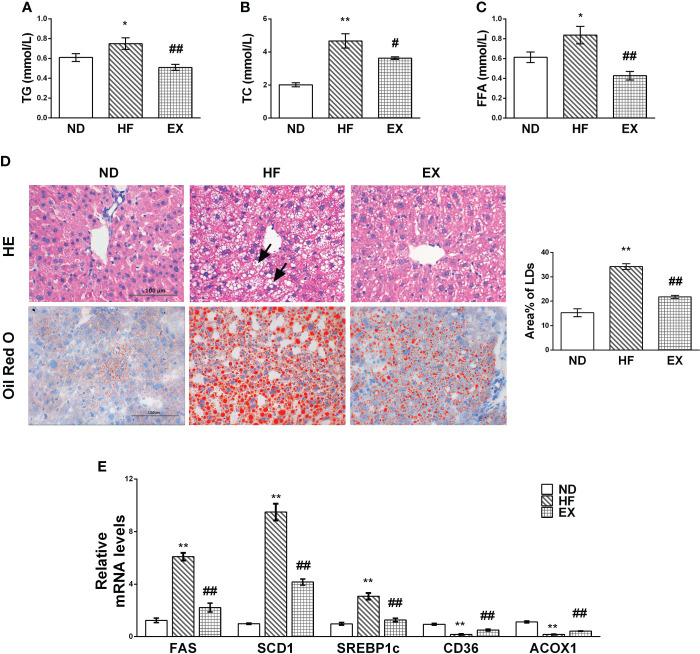
Mice with exercise training show improved lipid metabolism in serum and liver tissue samples. Male C57BL/6J mice were fed with a normal diet (ND) or a high-fat diet with (EX)/without (HF) swimming training for 4 months. Serum TG **(A)**, TC **(B)**, and FFA **(C)** levels were assessed (*n* = 5 per group). Representative images of liver sections at 400× magnification stained with HE (*n* = 3 per group), Oil Red O (*n* = 5 per group), and the average LD areas stained by Oil Red O **(D)**. Black arrowheads indicate hepatocellular swelling. Hepatic mRNA expression of FAS, SCD1, SREBP-1c, CD36, and ACOX1 by RT-PCR (*n* = 5 per group) **(E)**. *M* ± SEM; *P < 0.05 versus ND; **P < 0.01 versus ND; ^#^*P* < 0.05 versus HF; ^##^*P* < 0.01 versus HF.

No mice died throughout the duration of the experiment. We characterized the NAFLD in mice with 16-week high-fat diet and evaluated the lipid deposition in serum and liver, respectively. Increased FFA, TG, and TC were evident after 16 weeks of treatment in the HF group (**Figures 1A, B, C**). In contrast, the EX mice showed significantly lower levels of serum TG, TC, and FFA. As expected, a high-fat diet induced steatotic livers in mice, whereas exercise alleviated hepatic lipid accumulation, as assessed by the pathological characteristics in mice (**Figure 1D**). A significant difference was observed between ND and HF mice in the hepatic expression of lipogenic genes such as SREBP1c, SCD1, and FAS or lipid transport-related genes CD36 and fatty acid oxidation genes ACOX1 (**Figure 1E**). In contrast, this was reversed to varying degrees in the EX group, indicating that exercise reduces *de novo* lipid synthesis and enhanced fatty acid oxidation and uptake in the liver.

### Exercise reduces apoptosis and oxidative injury in mice on a high-fat diet

**Figure 2:**
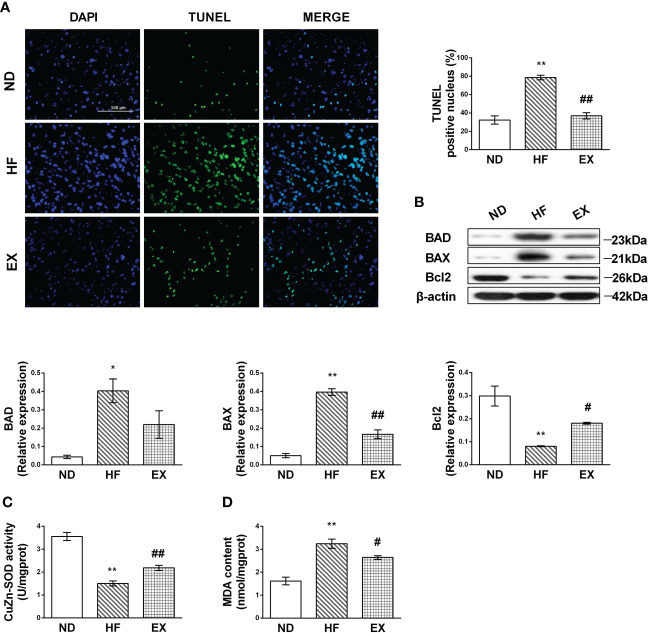
Exercise protects against hepatic lipotoxicity induced by 16 weeks of high-fat diet feeding. Representative images of liver sections stained with TUNEL **(A)** and the number of TUNEL-positive cells in liver sections (*n* = 3 per group). Hepatic protein expression of BAD, BAX, and Bcl2 **(B)** by Western blot analysis and graphs of their quantification. Quantification of immunoblots is shown in the graphs. (*n* =3 per group). CuZn-SOD activity and MDA content in the liver tissue sample **(C, D)**. *M* ± SEM; *P < 0.05 versus ND; **P < 0.01 versus ND; ^#^*P* < 0.05 versus HF; ^##^*P* < 0.01 versus HF.

As per previous studies (23**)**, we confirmed that chronic lipids and their derivatives deposit induced hepatocellular apoptosis in the liver. The HF mice fed with a 16-week high-fat diet showed a greater number of TUNEL-positive hepatocytes than normal-diet mice (**Figure 2A**). Western blotting revealed that the HF mice displayed higher levels of BAD and BAX and lower Bcl2 expression in the liver than the ND mice (**Figure 2B**). In contrast, these changes in apoptotic hepatocytes and the expression of apoptotic markers were remarkably inhibited by exercise. We also found that a 4-month high-fat feeding diet induced oxidative damage in the liver, indicated by increased MDA content and inhibited CuZn-SOD activity (**Figures 2C, D**). Exercise training reversed this incident.

### Exercise regulates hepatic MIF/MKK4/JNK expression in mice fed with a high-fat diet

**Figure 3:**
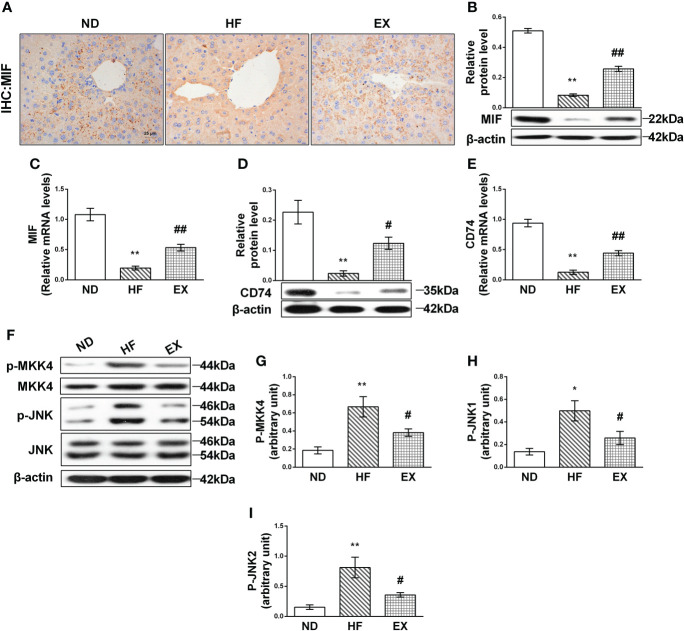
Effect of exercise on MIF-mediated JNK pathway in high-fat diet–induced NAFLD mice. Representative images of liver sections stained with MIF (*n* = 3 per group) **(A)**. Relative protein expression and mRNA levels of MIF **(B, C)** and CD74 **(D, E)** were measured by Western blot analysis (*n* = 3 per group) and qRT-PCR (*n* = 5 per group). MKK4 **(G)** and JNK **(H, I)** were assessed in homogenates from liver tissue by immunoblotting with phospho-specific and total antibodies (*n* = 3 per group). Quantification of immunoblots is shown in graphs **(A)**. *M* ± SEM. *P < 0.05 versus ND; **P < 0.01 versus ND; ^#^*P* < 0.05 versus HF; ^##^*P* < 0.01 versus HF.

To delineate the mechanism underlying exercise’s ability to alleviate liver lipotoxicity in HF mice, we validated MIF and its downstream mediated pathway. Immunohistochemistry showed that, in the HF group, there were fewer brown positive MIF deposits in the liver compared with the ND group; EX indicated relatively abundant MIF deposits (**Figure 3A**). RT-PCR and Western blot of MIF were then performed. Results showed that MIF proteins were significantly inhibited in liver tissues following 16 weeks of HFD in liver tissues (**Figure 3B**). Consistent with the protein results, the MIF mRNA content in HF-feeding liver tissue also displayed a marked decline (**Figure 3C**), indicating that HF-induced lipotoxicity correlates with decreased MIF expression of the liver. Notably, liver MIF significantly increased following the exercise treatment in mice compared with the control group, and these measurements correlated with the MIF receptor CD74, which was observed to be expressed consistent with MIF (**Figures 3D, E**).

The expression of JNK was then detected. As expected, JNK was highly activated by fatty acid in the HF group (**Figure 3F**). Of note, exercise restrained the activation of JNK1 and JNK2 (**Figures 3H, I**). Exercise also inhibited the expression of the phosphorylation of MKK4, which is the upstream kinase phosphorylate JNK and elevated in the HF liver (**Figure 3G**).

### JNK activation might contribute to liver lipotoxicity following lipid oversupply

**Figure 4:**
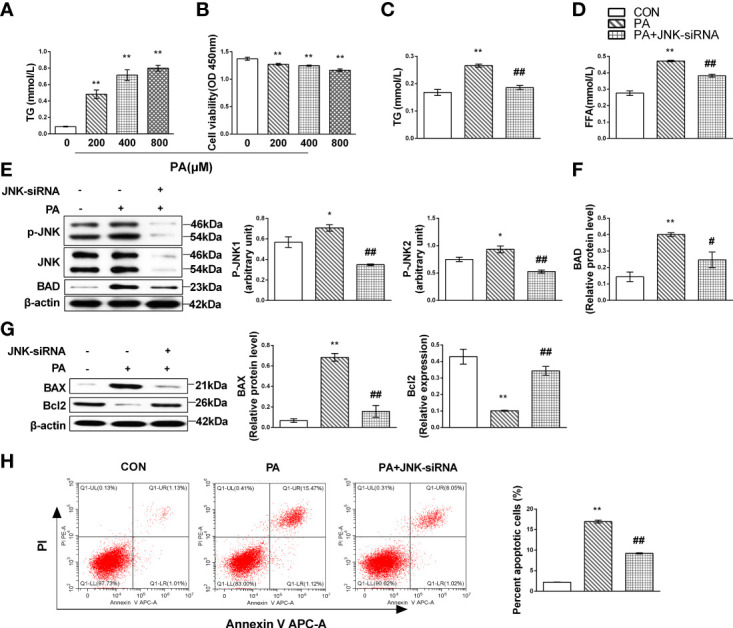
JNK activation might contribute to liver lipotoxicity following lipid oversupply. HepG2 cells were treated with the gradient concentrations of PA for 24 h; lipid accumulation was identified as cellular TG content and cytotoxicity by CCK-8 assays in the culture supernatant **(A, B)**. HepG2 cells were cultured with 400 mmol/L PA for 24 h and then transfected with JNK-siRNA. TG **(C)** and FFA **(D)** content were measured. Expression levels of JNK **(E)** and Bcl family proteins **(F, G)** by Western blot analysis. Apoptotic cells were identified by flow cytometry (*n* = 3 per each) **(H)**. Quantification of immunoblots is shown in the graphs below the blots (*n* = 3 per group). *M* ± SEM. *P < 0.05 versus Con; **P < 0.01 versus Con; ^#^*P* < 0.05 versus PA; ^##^*P* < 0.01 versus PA.

To determine whether hepatic MIF regulates JNK and mediates lipotoxicity, we first evaluated the relationship between JNK and lipotoxicity *in vitro* culture system with HepG2 cells treated with PA. We noted that the TG content accumulated and cell viability declined correspondingly with the cumulative concentration of PA (**Figures 4A, B**). We then transfected siRNA against JNK into HepG2 cells treated with PA and observed that FFA and TG decreased when JNK was inhibited (**Figures 4C, D**). Both JNK1 and JNK2 protein levels, which were activated in PA-treated cells, were limited (**Figure 4E**). We also performed tests of apoptotic proteins Bcl2 family and JNK knockdown attenuated hepatocellular apoptosis as evidenced by decreases in and expression in PA-supplemented HepG2 cells (**Figures 4F–H**).

### rMIF regulates MKK4/JNK pathway in HepG2 cells with or without PA cultivation

**Figure 5:**
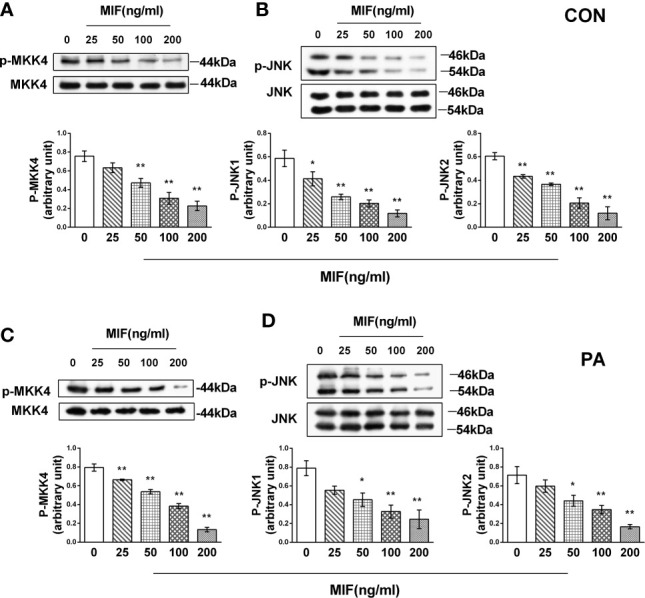
rMIF supplementation regulates MKK4/JNK pathway in PA-treated or not HepG2 cells. Gradient concentrations of rMIF (0, 25, 50, 100, 200 ng/ml) were used to stimulate HepG2 cells for 24 h Protein expression levels of phosphorylated and total MKK4 **(A)** and JNK **(B)** were detected by Western blot analysis. HepG2 cells were administrated with 400 mmol/L PA for 24 h and then cultured with the indicated concentration of rMIF, phosphorylated, and total protein expression of MKK4 **(C)** and JNK were measured **(D)** (*n* = 3 per group). Quantification of immunoblots is shown in the graphs below the blots. *M* ± SEM. *P < 0.05 versus Con; **P < 0.01 versus Con.

When HepG2 cells were administrated with different concentrations of rMIF, the phosphorylation of both JNK1 and JNK2, which are mainly distributed in the liver, was inhibited by MIF in a dose-dependent manner. Consistent with the decrease in JNK phosphorylation, the phosphorylation of MKK4, upstream of JNK, also reduced after rMIF supplementation (**Figures 5A, B**). Notably, an identical tendency was observed in the PA-treated HepG2 cells (**Figures 5C, D**).

### MIF regulates MKK4/JNK and alleviated lipid accumulation in PA-treated HepG2 cells

To determine the relationship between lipotoxicity and MIF expression, HepG2 cells were treated with rMIF and MIF siRNA followed by PA supplementation.

**Figure 6:**
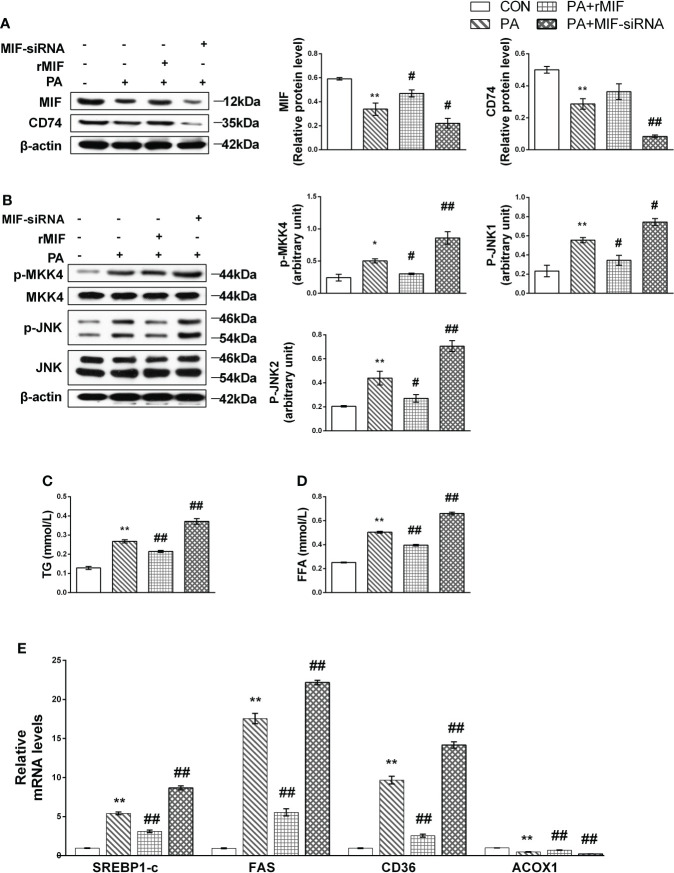
MIF inhibits MKK4/JNK and alleviated lipid accumulation in PA-treated HepG2 cells. HepG2 cells were cultured with 400 mmol/L PA for 24 h, and then exogenous rMIF 200 ng/ml was added for 24 h or transfected with MIF siRNA; protein expression levels of MIF and CD74 **(A)**, phosphorylated, and total MKK4 and JNK **(B)** were detected by Western blot analysis (*n* = 3 per group). Quantification of immunoblots is shown in the graphs below the blots. mRNA expression of FAS, SREBP-1c, CD36, and ACOX1 were detected by RT-PCR **(C, D)** (*n* = 5 per group). TG and FFA contents were measured **(E)**. (*n* = 3 per group) *M* ± SEM. *P < 0.05 versus Con; **P < 0.01 versus Con; ^#^*P* < 0.05 versus PA; ^##^*P* < 0.01 versus PA.

At first, we evaluated the protein levels of the MIF/JNK pathway. Under the animal experiment, the protein expression level of MIF and its receptor CD74 decreased after being PA cultured (**Figure 6A**); simultaneously, JNK and MKK4 phosphorylation in PA-induced cells was significantly higher than that in the control group (**Figure 6B**).

rMIF supplementation attenuated the phosphorylation of MKK4 and JNK, accompanied by TG and FFA content reduced to varying degrees. The administration of the MIF siRNA completely suppressed MIF expression and showed deteriorated performance of lipid accumulation (**Figures 6C, D**) and metabolism disorder (**Figure 6E**). Conversely, JNK phosphorylation was promoted after the replenishment of MIF siRNA.

### MIF regulates JNK-mediated lipotoxicity in PA-treated HepG2 cells

**Figure 7:**
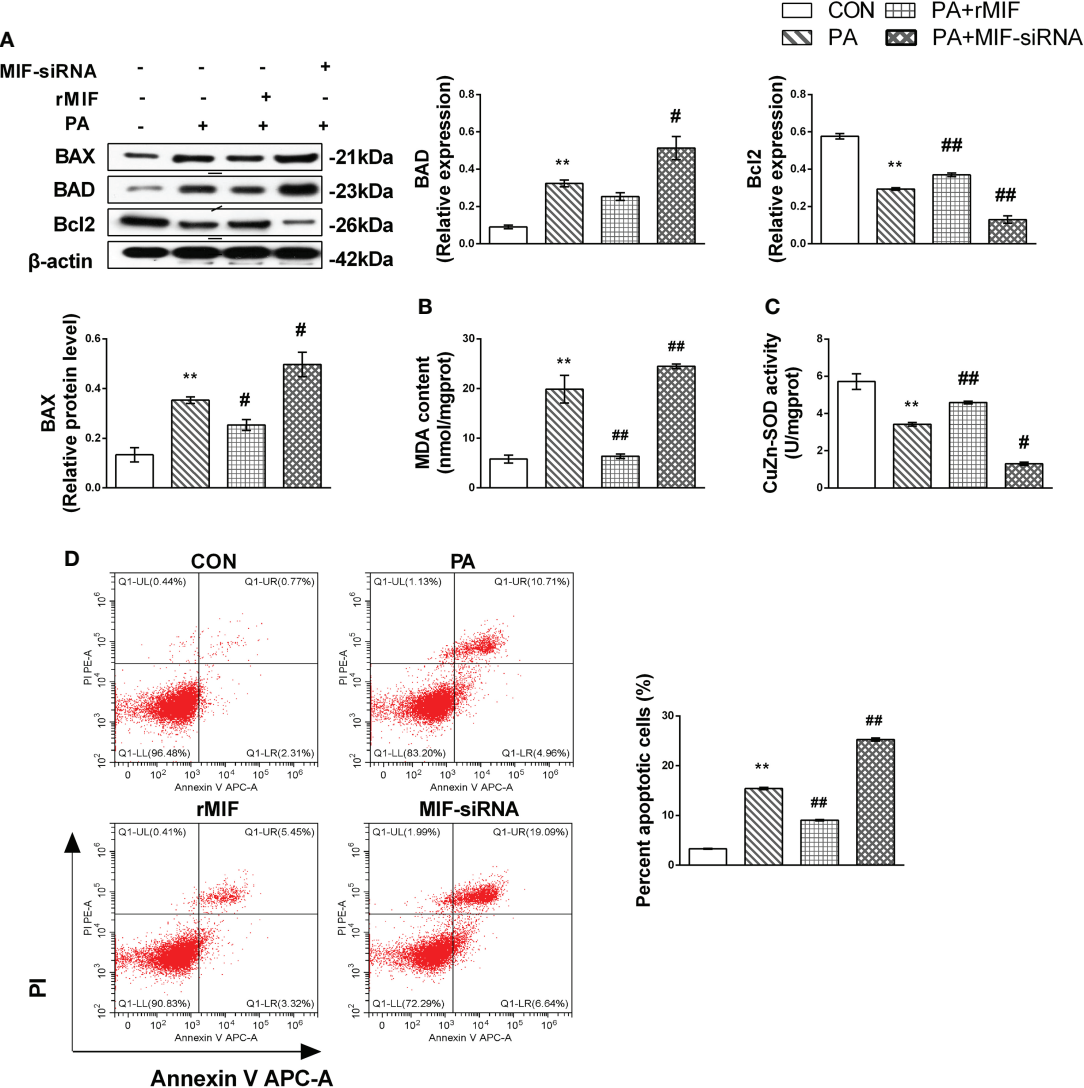
MIF regulates JNK-mediated lipotoxicity in PA-treated HepG2 cells. HepG2 cells were cultured with 400 mmol/L PA for 24 h and then added exogenous rMIF 200 ng/ml for 24 h or transfected with MIF siRNA; protein expression levels of BAD, BAX, and Bcl2 **(A)** were detected by Western blot analysis. CuZn-SOD activity and MDA contents were measured **(B, C)**. Quantification of immunoblots is shown in the graphs below the blots. Apoptotic cells were identified by flow cytometry (*n* = 3 per each) **(D)**. (*n* = 3 per group) *M* ± SEM. *P < 0.05 versus Con; **P < 0.01 versus Con; ^#^*P* < 0.05 versus PA; ^##^*P* < 0.01 versus PA.

Finally, we examined cellular apoptosis and injury to further delineate the effects of MIF expression on lipotoxicity in HepG2 cells. The results indicated that PA induced prominent apoptosis (**Figures 7A, D**) in HepG2 cells. Nevertheless, this effect was reversed by treatment with rMIF, which presented lower expression of pro-apoptotic protein and fewer apoptotic cells. The addition of rMIF also reduced oxidative injury induced by PA (**Figures 7B, C**). However, the condition of apoptosis and injury deteriorated following the administration of MIF siRNA.

## Discussion

Here, we observed that, after an extended period of high-fat feeding, mice had an immediate elevated FFA in circulation and synthesis in the liver. It is uncertain whether exercise reduced lipid profiles through increasing hepatic FFA uptake and oxidation (24), or these changes in the liver are dependent on the improvement of blood FFA levels resulting from exercise because transferred FFAs to the liver are mainly derived from the plasma. The improved lipid profiles may also be an overall outcome integrated by other organs and corresponding physiological mechanisms such as the efficiency of adipose tissue, triglyceride lipolysis, or skeletal muscle utilization (25).

PA is the most common saturated FFA involved in the event of NAFLD (26). Numerous *in vitro* studies have demonstrated that the toxic effects of PA induce apoptosis on hepatocytes (27, 28). We observed that the vitality of cells declined when cultured with an incremental dose of PA, which verifies its toxicity in HepG2 cells. Apoptotic cells appear to be central to the pathogenesis of lipotoxic injury in the liver (2). Our results indicated that cell apoptosis following lipid oversupply can be partly reversed by exercise. The mice in our EX group received a high-fat diet and exercise training concurrently, which may reveal the preventive impact of exercise on hepatic lipotoxicity. Increased active products are also considered to be an important toxicological manifestation of excess lipid in the liver (29). The excess FFAs retard the mitochondrial oxidation efficiency; generated reactive oxygen can lead (30) to metabolism disorder (31), cell injury, or even death (32).

MIF has been reported to inhibit apoptosis in myocardium (33), cervical cancer cells (34), neuroblastoma cells (35), and multiple myeloma cells (36). The cytokine activity of MIF, as well as its protective effects on hepatocytes, is largely due to its binding to the main receptor CD74 (37). Although recent studies have illustrated that MIF-CD74 signaling is protective in fatty liver injury by enhancing pro-survival pathways (9), a complex interaction exists between MIF and the progression of liver disease. MIF elicits detrimental effects at specific phases and models of liver disease, and studies have raised that MIF contributes to ethanol-induced hepatocyte damage (38, 39); however, none have reported on how MIF reacts under hepatic lipotoxicity. The results presented here indicate that MIF is downregulated in diet-induced steatotic livers (long-term high-fat feeding induced decreased expression of MIF) as well as its receptor CD74. Specifically, we observed increased activation of the JNK pathway and toxicity following lipid oversupply. These effects were reversed by exercise to a significant extent. This may be owed to the autocrine action of MIF in the liver, since it was found that MIF suppressed the JNK pathway activation in HepG2 cells alone and after PA treatment. The activation effect of MIF by exercise in the normal livers has been previously studied (16). We propose that this activation effort may occur in fatty liver as well and has relevance to lipotoxicity following extensive lipid infiltration.

Previous evidence has shown that exercise increases the expression of MIF and mediates neuroprotection in rodent models (40, 41); our results, in agreement with Moon’s research (42), indicate that MIF is regulated by exercise to prevent hepatic steatosis. Nevertheless, the specific mechanism by which exercise regulates MIF remains unclear. It has been reported that hypoxia-inducible factor-1 (HIF-1), which is the body adaptation regulator during exercise and expressed highest in the liver (19), can regulate the secretion and release of MIF (43). HIF-1 was found to be involved in tissue hypoxia redistribution during exercise and systematic metabolic homeostasis after exercise in the liver, which may contribute to the regulation effect of exercise on MIF in the liver (44).

MKK4 is the primary upstream JNK kinase that is activated during acute liver injury (45) and regulated by MIF in the heart (14). In the context of previous studies, we provide further evidence that HFD enhanced the phosphorylation of MKK4 and JNK protein levels in mice with NAFLD (46). Consistent with JNK, the elevated MKK4 in the liver after high-fat diet was inhibited by exercise. However, the absence of endogenous hepatic MIF in PA cultured HepG2 cells led to increased MKK4 activation, which is likely responsible for the excess activation of the JNK pathway. Similarly, the addition of MIF inhibits the activation of MKK4, which suggests that exercise potentially affects MIF and modulates the phosphorylation of MKK4/JNK during lipotoxicity. In *in vitro* experiments, we confirmed that the excess MIF supplementation suppressed the expression of MKK4 and JNK in HepG2 cells with or without PA culture, which is consistent with the previous findings in myocardium (14). We further observed that MIF improved lipid accumulation and metabolism, as well as reduced cell death and apoptotic protein expression. In contrast, following MIF inhibition, lipid infiltration and apoptosis were aggravated. These observations are consistent with the findings that MIF^-^/^-^ mice fed with HFD exhibit enhanced hepatic fatty degeneration and lipid accumulation (10). Our data suggest that MIF plays an important role in regulating hepatocellular injury and lipotoxicity.

**Figure 8:**
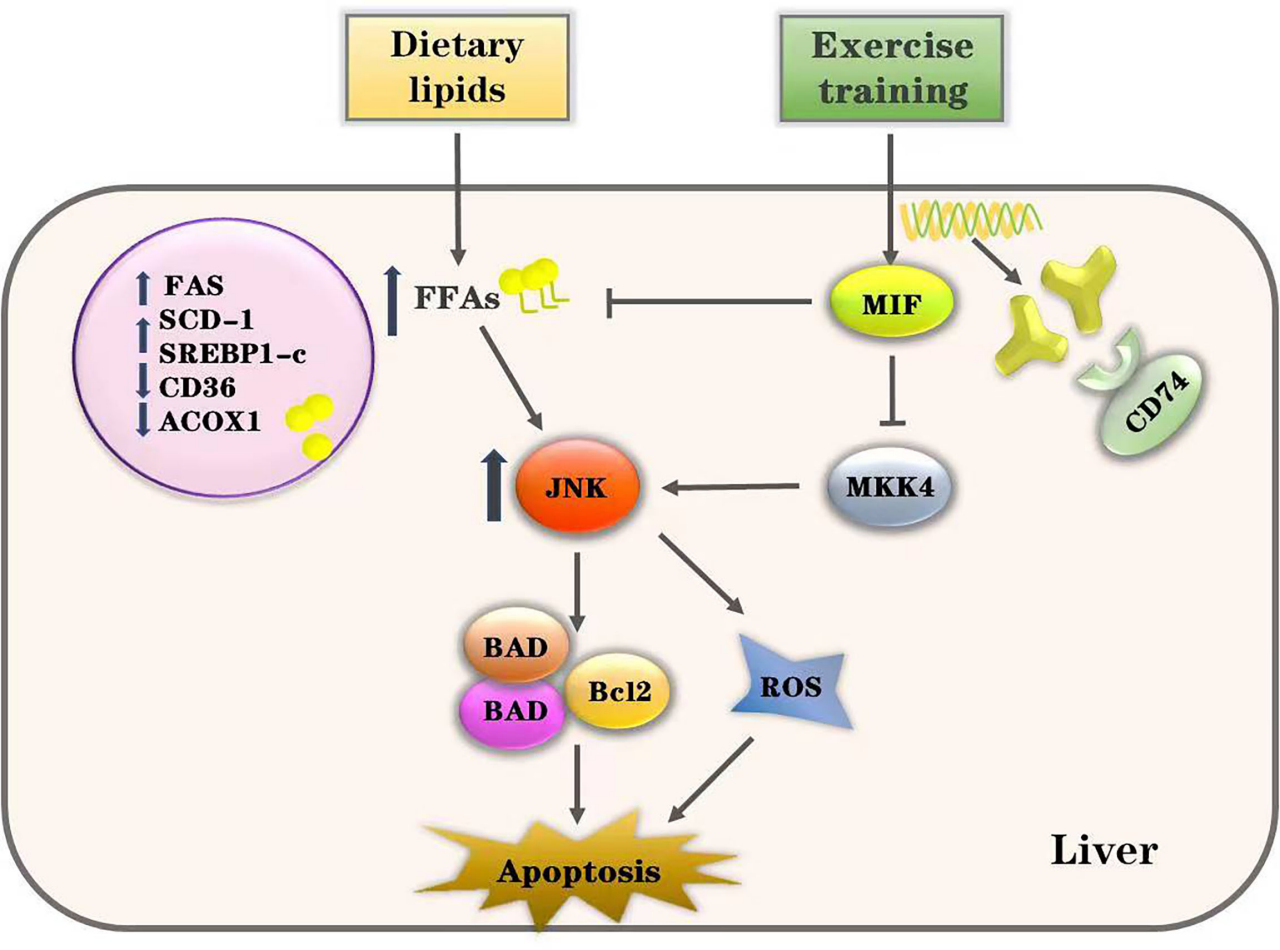
Proposed pathway of exercise’s effects on HFD-induced lipotoxicity. Excessive intake of dietary lipids induced metabolic disorders of free fatty acids (FFAs) and induced the activation of JNK-mediated apoptosis in the liver. However, our data show that exercise training alleviates hepatic lipotoxicity and suppresses the JNK pathway through enhancing MIF signaling. Thus, we conclude that exercise inhibits JNK pathway activation and lipotoxicity *via* MIF in NAFLD.

JNK has been widely established in mediating damage and apoptosis in various tissues (47–49), appearing to play an important role in the development of lipotoxicity. The ultimate effect of JNK in lipotoxicity is to upregulate pro-apoptotic BH3-ONLY protein and inhibit the expression of anti-apoptotic protein BCL2/BCLXL (50), leading to ROS production and apoptosis (51, 52). Saturated fatty acids activate Bcl2 family proteins such as BAD and BAX through the JNK pathway and directly induce hepatocyte apoptosis (53, 54). Our results show that enhanced JNK activation is associated with the increased hepatocyte apoptosis. Phosphorylation of JNK in the liver was significantly activated after HFD treatment in mice and PA-cultured cells. We found that JNK pathway inhibition improved the expression of apoptotic proteins and reduced cell death in PA-cultured HepG2 cells. JNK inhibition is known to have decreased lipid deposition and improved hepatocyte apoptosis in NAFLD (55). It is suggestive that endogenous hepatic MIF suppresses the activation of the JNK pathway during lipotoxicity through the action of its receptor, CD74. The protective effect of exogenous rMIF in HepG2 cells was likely due to its suppression of excessive JNK pathway activation (**Figure 8**).

There are certain limitations in this research. Immortalized hepatocyte models have been widely used in hyperglycemia or lipid cultured cell models, which parallel the NAFLD (56) owing to their availability, similarity, and high overlap with primary hepatocytes (57); however, more studies are necessary to elucidate the role of MIF in other cell lines and primitive hepatocytes. Another limitation was that only male mice were involved in this study. It was reported that the incidence and severity of NAFLD in men are higher than in women under the age of 50 years (58). Animal experiments also showed that male mice tend to have more severe liver steatosis compared with female mice (59). However, women have a higher risk of advanced fibrosis than men after 50 years (60). Moreover, how sex difference influences the response of NAFLD patients to exercise remains unclear and inconsistent (61) (62). Therefore, further consideration of sex differences in future investigations on NAFLD are needed to clarify the efficacy of exercise intervention.

To conclude, we found that exercise reduces lipotoxicity and inhibits JNK activation possibly through modulating endogenous hepatic MIF in NAFLD. Its clinical implications aid in the understanding of exercise’s role in the prevention of NAFLD and lead a step toward developing strategies in interventions for patients with high-fat diet evoked NAFLD.

## Data availability statement

The original contributions presented in the study are included in the article/**Supplementary Material**. Further inquiries can be directed to the corresponding author.

## Ethics statement

This study was reviewed and approved by the Animal Ethics Committee of Xiangya Medical School, Central South University.

## Author contributions

NC, LH designed and performed parts of the study and wrote the original draft supervised by SL. YD, BY, DL,YL, LQ, CL provided assistance in coordinating the study and aided in data collection. JR-G was involved in writing - review and editing, visualization. SL and YD were involved in securing funding for the study. SL, supervised the project and administration and had the final responsibility for the decision to submit for publication. All authors contributed to the article and approved the submitted version.

## Funding

This work was supported by grants from the National Natural Science Foundation of China (Grant number: 82172549 to SL and 82002403 to YD), and Natural Science Foundation of Hunan Province (Grant number: 2021JJ70073 to SL and 2021JJ40981 to YD).

## Conflict of interest

The authors declare that the research was conducted in the absence of any commercial or financial relationships that could be construed as a potential conflict of interest.

## Publisher’s note

All claims expressed in this article are solely those of the authors and do not necessarily represent those of their affiliated organizations, or those of the publisher, the editors and the reviewers. Any product that may be evaluated in this article, or claim that may be made by its manufacturer, is not guaranteed or endorsed by the publisher.
