# Supplementary material for: Exercise inhibits JNK pathway activation and lipotoxicity via macrophage migration inhibitory factor in nonalcoholic fatty liver disease
Source: Front Endocrinol (Lausanne). 2022 Sep 6;13:961231. doi: 10.3389/fendo.2022.961231 (PMC9485555; doi:10.3389/fendo.2022.961231)
Supplement: Supplementary file 1 [file Table_1.docx]

Supplementary Table 1

Sequence of primers used for RT-qPCR assays in mice

| Genes | Sequence | Species | Suppiler |
| --- | --- | --- | --- |
| MIF | F-CATGACTTTTAGCGGCACGAACGA  R-ACCACCGATCTTGCCGATGCTG | Mouse | Sangon Biotech |
| FAS | F-ACCATCAGAAATTCAGCCCGTT  R-CCCTGCAATTTCCGTTTGGCTT | Mouse | Sangon Biotech |
| SREBP-1c | F-CGCAAGGCCATCGACTACATCCG  R-CCTCCACTGCCACAAGCTGACAC | Mouse | Sangon Biotech |
| CD36 | F-AAGTTGCCATAATTGAGTCCT  R-CTTTAAGGTCGATTTCAGATCCG | Mouse | Sangon Biotech |
| CD74 | F-GTCAGCCACATCCCTGCCGTCT  R-AGTTCCTGCCTGGTCACTCCC | Mouse | Sangon Biotech |
| SCD1 | F-ATCCCATCGCCTGCTCTACCC  R-AAACCTGCCCTCCTGACTCTCG | Mouse | Sangon Biotech |
| ACOX1 | F-ATGCCTTTGTTGTCCCTATCCG  R-ATTGTCCATCTTCAGGTAGCCAT | Mouse | Sangon Biotech |
| β-actin | F-ACATCCGTAAAGACCTCTATGCC R-TACTCCTGCTTGCTGATCCAC | Mouse | Sangon Biotech |

Supplementary Table 2

Sequence of primers used for RT-qPCR assays in cells

| Genes | Sequence | Species | Suppiler |
| --- | --- | --- | --- |
| FAS | F-CGTCTGTTGCTAGATTATCGTCC  R-CTGTGCAGTCCCTAGCTTT | Human | Sangon Biotech |
| SREBP-1c | F-TCCCAGCCCCTCAGATACCAC  R-CCCATTGAGCAGCCAGACCAC | Human | Sangon Biotech |
| CD36 | F-AAAATGTAACCCAGGACG  R-GTGTCGATTATGGCAACT | Human | Sangon Biotech |
| ACOX1 | F-CTCCCTCGATGCGGAGTCA  R-GCTGGAAGTCTGGGTCGTT | Human | Sangon Biotech |
| Si-MIF | GCTGGACAACTCCACCTT | Human | Ribobio |
| Si-JNK | CACCAAAGATCCCTGACAA | Human | Ribobio |
| β-actin | F- ACCCTGAAGTACCCCATCGAG  R- AGCACAGCCTGGATAGCAAC | Human | Sangon Biotech |

Supplementary Table 3

| Antibodies | Supplier | Catalog |
| --- | --- | --- |
| BAD | Abcam | 32445 |
| BAX | Abcam | 32503 |
| BCL2 | Abcam | 182858 |
| phospho-JNK(Thr183/Tyr185) | Cell Signaling Technology | 4668 |
| JNK | Cell Signaling Technology | 9252 |
| phospho-MKK4(Ser257) | Cell Signaling Technology | 4514s |
| MKK4 | Cell Signaling Technology | 9152s |
| CD74 | Santa Cruz | 6262 |
| MIF | Proteintech | 20415-1-AP |
| β-actin | Proteintech | 66009-1-Ig |
